# Spectrum of *EGFR* Gene Copy Number Changes and *KRAS* Gene Mutation Status in Korean Triple Negative Breast Cancer Patients

**DOI:** 10.1371/journal.pone.0079014

**Published:** 2013-10-30

**Authors:** Yoonjung Kim, Juwon Kim, Hy-De Lee, Joon Jeong, Woochang Lee, Kyung-A Lee

**Affiliations:** 1 Department of Laboratory Medicine, Yonsei University College of Medicine, Seoul, Republic of Korea; 2 Department of Laboratory Medicine, Yonsei University Wonju College of Medicine, Wonju, Republic of Korea; 3 Department of Surgery, Yonsei University College of Medicine, Seoul, Republic of Korea; 4 Department of Laboratory Medicine, University of Ulsan College of Medicine and Asan Medical Center, Seoul, Republic of Korea; University of Nebraska Medical Center, United States of America

## Abstract

Anti-epidermal growth factor receptor (EGFR) therapy has been tried in triple negative breast cancer (TNBC) patients without evaluation of molecular and clinical predictors in several randomized clinical studies. Only fewer than 20% of metastatic TNBCs showed response to anti-EGFR therapy. In order to increase the overall response rate, first step would be to classify TNBC into good or poor responders according to oncogenic mutation profiles. This study provides the molecular characteristics of TNBCs including *EGFR* gene copy number changes and mutation status of *EGFR* and *KRAS* gene in Korean TNBC patients. Mutation analysis for *EGFR, KRAS, BRAF* and *TP53* from a total of 105 TNBC tissue samples was performed by direct sequencing, peptide nucleic acid-mediated PCR clamping method and real-time PCR. Copy number changes of *EGFR* gene were evaluated using multiplex ligation-dependent probe amplification. Out of all 105 TNBCs, 15.2% (16/105) showed *EGFR* copy number changes. Among them, increased or decreased EGFR copy number was detected in 13 (5 single copy gain, 2 amplification and 4 high-copy number amplification) and 3 cases (3 hemizygous deletion), respectively. The mutation frequencies of *KRAS*, *EGFR* and *TP53* gene were 1.9% (G12V and G12D), 1.0% (exon 19 del) and 31.4%, respectively. There was no *BRAF* V600E mutation found. Future studies are needed to evaluate the clinical outcomes of TNBC patients who undergo anti-EGFR therapy according to the genetic status of *EGFR*.

## Introduction

Triple-negative breast cancers (TNBCs) are tumors, nominally classified as a diagnosis of exclusion, that do not express clinically significant levels of the estrogen receptor (ER), progesterone receptor (PgR) and epidermal growth factor receptor 2 (Her-2) over-expression or gene amplification [1]. TNBCs account for 10–24% of all invasive breast cancers with a strong predilection for young women, but the frequency varies by race and in some racial groups reaches a frequency of up to 55% [2]–[6]. Hormonal therapies and HER2-targeted agents are not effective in these breast tumors, which tend to exhibit aggressive, metastatic behavior and have a worse prognosis than hormone receptor-positive, luminal subtypes [1], [7], [8]. In primary non–small cell lung cancer that harbors *EGFR* mutations, multiple randomized clinical trials comparing first-line chemotherapy to EGFR tyrosine kinase inhibitors (TKIs) have been performed and uniformly demonstrated the superiority of EGFR-TKIs [9]–[12]. In addition, patients suffering from recurrent glioblastoma with EGFR amplification and those lacking EGFRvIII expression have been treated with the EGFR-targeted monoclonal antibody cetuximab with a significantly superior progression-free and overall survival [13]. Approximately 20% of metastatic TNBCs showed response to anti-EGFR therapy in randomized clinical trials [2], [14]. Recent studies have shown no mutations in several target genes associated with the receptor tyrosine kinase/RAS/MAPK pathway, including *EGFR, KRAS* and *BRAF*, in the absence of HER2 gene amplification [15]–[17]. However, in Asians, *EGFR* mutations and copy number changes of the *EGFR* gene were detected in up to 11.4% [17], [18] and 21% of TNBCs [19], respectively. Anti-EGFR therapies are still an attractive treatment modality according to the genetic profiles of TNBCs [18]–[20]. Thus, it would be beneficial to evaluate mutations and copy number changes of *EGFR* in TNBC patients before treating with anti-EGFR drugs, which in turn would improve the response rates compared to previous data. Furthermore, a deliberate and clinically applicable method is also needed to evaluate EGFR mutations and copy number changes as a molecular predictor for the patients. Here, we report the mutation status of *EGFR*, *KRAS* and *BRAF*, and the frequency of *EGFR* copy number changes in Korean patients with TNBCs.

## Materials and Methods

### Subject selection

We obtained a total of 105 tissue samples from TNBC patients at the time of surgery. Triple negative status (negative estrogen receptor (ER), progesterone receptor (PgR) and c-erbB2) of the tumors was confirmed by immunohistochemical (IHC) staining. Briefly, all IHC staining was performed using formalin-fixed, paraffin-embedded tissue sections. After deparaffinisation/rehydration and antigen retrieval, paraffin sections were incubated with primary antibodies against ER (1∶50 dilution; Dinona, Seoul, Korea), PR (1∶100 dilution; Dinona) and Her2/neu (1∶250 dilution; Dako, Glostrup, Denmark). ER and PR IHC signal was evaluated using the Allred score [21]. A score of 0 to 2 was considered negative and a score of 3 to 8 was regarded as positive. HER2 status was determined by IHC using the HercepTest, and score of 0–1+ was regarded as negative (18). A borderline/equivocal expression of HER-2 was indicated for cerb2 when at least 10% of tumor cells demonstrated 2+ cytoplasmic membrane staining, and these samples were confirmed using fluorescence *in situ* hybridization with the PathVysion HER2 DNA Probe kit (Abbott, IL, USA) according to the manufacturer instructions. A HER2 gene-to-chromosome 17 ratio greater than 2 was considered positive. The study was approved by the Institutional Review Board of the Gangnam Severance Hospital and written informed consent was obtained from the patients.

### DNA preparation

DNA was extracted from breast cancer tissues (ER-, PR-, and HER2-) obtained at the time of surgical resection. Genomic DNA was extracted using QIAamp DNA extraction kit (Qiagen, Hilden, Germany) according to the manufacturer protocol. The concentration and quality of genomic DNA was evaluated by Nanodrop (ND-1000; Thermo Scientific, DE, USA).

### Direct sequencing of *EGFR*, *KRAS* and *TP53* genes

Mutation analysis for *EGFR* and *KRAS* genes was performed in duplicate using direct sequencing and the peptide nucleic acid (PNA)-mediated PCR clamping method. PCR amplification and direct sequencing of *EGFR* gene (exons 18–21), *KRAS* (exon2) and *TP53* gene (exon 5–9) were performed in 105 TNBCs [22]–[27]. The primers designed to amplify exons and flanking introns of those genes are summarized in Table 1. PCR was performed using an Accu-Power™ Premix (Bioneer, Daejeon, Korea) under the following amplification conditions: 94°C for 4 min followed by 50 cycles of 94°C for 1 min, 60°C for 30 s and 72°C for 30 s, and final extension at 72°C for 15 min. Purified PCR products obtained using a QIAquick Gel Extraction kit (Qiagen, Düsseldorf, Germany) were used for sequencing with a Big Dye Terminator Cycle Sequencing Ready Reaction kit (Applied Biosystems, Foster City, CA, USA). The thermal cycler conditions were as follows: 96°C for 5 min followed by 24 cycles of 96°C for 10 s, 50°C for 5 s and 60°C for 4 min, and final extension at 72°C for 5 min. The sequences were analysed using ABI 3500Dx system (Applied Biosystems). Sequences were compared with the database sequence in GenBank (http://www.ncbi.nlm.nih.gov assessed June, 2012). The GenBank accession numbers are NM_005228.3, NM_004985.3 and NC_000017.9 for the *EGFR, KRAS* and *TP53* genes, respectively.

**Table 1:** PCR primers of *TP53* and *EGFR* gene.

Target	Exon	Forward Primer (5'→3')	Reverse Primer (5'→3')	PCR product size (bps)
*EGFR*	18	ATGTCTGGCACTGCTTTCCA	ACAGCTTGCAAGGACTCTGG	277
	19	AGATCACTGGGCAGCATGT	AGCAGCTGCCAGACATGAG	246
	20	CATTCATGCGTCTTCACCTG	CATATCCCCATGGCAAACTC	377
	21	AGCCATAAGTCCTCGACGTG	ATCCTCCCCTGCATGTGTTA	372
*KRAS*	2	TGTATTAACCTTATGTGTGACA	CTTGTAATAAGTACTCATGAAA	279
*TP53*	5&6	CACTTGTGCCCTGACTTTCA	TTGCACATCTCATGGGGTTA	619
	7	CCTGCTTGCCACAGGTCT	TGATGAGAGGTGGATGGGTAG	279
	8&9	CAAGGGTGGTTGGGAGTAGA	CCCCAATTGCAGGTAAAACA	496

Abbreviations: bps, base pairs; PCR, polymerase chain reaction.

### Mutation analysis of *EGFR, KRAS* and *BRAF* genes

We evaluated *EGFR* and *KRAS* mutations using the PNAClamp™ *EGFR* and PNAClamp™ *KRAS* Mutation Detection kits (Panagene, Inc., Daejeon, Korea). PNA-mediated PCR clamping is a mutant enrichment method and can detect minor mutant alleles in whole tumor cells [14], [28], [29]. All reactions were carried out in a volume of 20μl with template DNA, a primer and PNA probe set, and SYBR Green PCR master mix according to the manufacturer instructions. Real-time PCR reaction was performed using a CFX 96 (Bio-Rad, USA). PCR cycling conditions were as follows: 5 minutes at 94°C followed by 40 cycles of 94°C for 30 seconds, 70°C for 20 seconds, 63°C for 30 seconds, and then 72°C for 30 seconds. Each kit is designed to detect 29 mutations in the *EGFR* gene (exon 18 – 21) and 7 mutations in the *KRAS* gene (exon 2). Delta *C_t_* values were calculated for defining whether there were mutations in *EGFR* and *KRAS* [28], [29]. *BRAF*V600E was evaluated using Real-QTM™ BRAF V600E detection kit (Biossewoom, Inc., Seoul, Korea). All optical reaction tubes which contained 10 µL genomic DNA and 15 µL RQ-PCR reaction mixture were placed in a CFX96 (Bio-Rad, USA). The thermal cycler protocol used was 50°C for 120 minutes, 95°C for 10 minutes and then 40 cycles of 95°C for 15 seconds and 58°C for 45 seconds. Data analysis was done according to the manufacturer instructions.

### Copy number analysis of *EGFR*

*EGFR* copy number changes in TNBCs were evaluated using SALSA® MLPA® Probemix P315-B1 EGFR kit (MRC-Holland, Amsterdam, the Netherlands) that contains 12 reference probes and 30 probes in the exons of the *EGFR* gene, including specific probes for the L858R and T790M mutations. Probe sequences of SALSA® MLPA® P315-B1 EGFR probemix are available at http://www.mlpa.com. Denatured DNA samples were hybridized with probemix for 16 hours at 60°C. Ligation of probes was performed with the Ligase-65 enzyme at 54°C for 15 min, followed by 5 minutes at 98°C for heat inactivation of the Ligase-65 enzyme. PCR was performed with the specific SALSA PCR primers for 35 cycles (95°C for 30 s, 60°C for 30 s and 72°C for 1 min) using the GeneAmp® PCR System 9700 (Applied Biosystems, USA). All MLPA analysis of 105 samples was performed in duplicate. Three cancer cell lines (HCC827, H2279, and H1975) were used as the positive controls for MLPA analysis. According to results from fluorescence in situ hybridization and/or the nanofluidic digital PCR array, HCC827, H2279 and H1975 were considered to have high-copy number amplifications, amplifications and single copy gain with L858R and T790M mutations, respectively [30], [31]. To determineanalytical sensitivity of MLPA for EGFR copy number changes in tumor cells, DNA from HCC827 was serially diluted with wild type DNA. Each spiked sample was assayed up to five times using SALSA®MLPA® Probemix P315-B1 EGFR kit (MRC-Holland).

MLPA fragment analysis data were generated on Applied Biosystems 3500Dx system (Applied Biosystems). The data were analyzed using the GeneMarker® software (SoftGenetics, PA, USA). The copy number of the *EGFR* gene was determined on the basis of the relative peak height ratio of the probes. Seventy-eight good quality samples (the SD of all 44 probes were within 0.1) without gain and/or loss were used as a reference group to construct the mean peak of each probe. The SD of all control probes in 105 samples, except by random errors due to capillary electrophoresis conditions (ex. SPG11 gene, 439 nt, 16538-L21570) and disease-associated control probe (ex.COL11A1 gene, 196 nt, 13232-L14565 etc.) [32], was within 0.1. Based on our previous report with MLPA copy number detection, we used thresholds of 1.2 and 0.8 for the detection of single copy gain (GAIN) and hemizygous deletion (HETD), respectively. Furthermore, the ratios above 2.0 were considered as amplifications (AMP) and those exceeding 10 as high-copy number amplifications (HLAMP) [33]. The probe fractions are then divided by the average probe fraction of the reference group, resulting in a probe ratio [34].

### Statistical Analysis

Patient follow-up periods were calculated as time between surgery date and the date of last follow-up (months). Relapse free survival (RFS) included loco-regional recurrence, distant metastasis, and death from any cause. Breast cancer-specific survival (BCSS) included only patients who died from any breast cancer-related cause. TP53 mutations were classified as missense, nonsense, and frameshift mutations. In addition, missense mutations were further subdivided as missense mutations in DNA binding motifs (DBM) and outside the DBM according to mutation position, nature, and suspected effect on protein structure and activity [23], [35]. Kaplan-Meier survival curves with log-rank tests were performed to compare RFS and BCSS according to the type of TP53 mutation. Cox proportional hazard models were performed to assess the influence of prognostic factors on RFS. Univariate analyses for age at surgery, tumor size, node status, Ki67 labeling index and TP53 mutations were performed. Any factors from the univariate analysis with a *p* value less than 0.10 was included in the multivariate analysis. All statistical analyses were performed using SPSS 20.0 (SPSS Inc, Chicago, IL, USA). For all statistical analyses except the univariate analysis, a *p* value less than 0.05 was regarded significant.

## Results

### Patient characteristics

The clinicopathological characteristics of TNBCs are listed in Table 2. The median age of patients was 49 years old (range, 28–76) and the median tumor size was 22 mm (range, 5–100 mm). The majority of TNBCs (84/105, 78.5%) were invasive ductal carcinomas with nuclear grade 2–3 and histologic grade II-III. Of 105 TNBCs, 33 (31.4%) carried mutations in exon 5–9 of the *TP*53. Sixteen (48.5%) carried a missense mutation, and 17 (51.5%) harbored a frameshift, nonsense, or splicing mutation.

**Table 2:** Clinicopathological Characteristics of Triple Negative Breast Cancers (n = 105).

		Numbers	Percentage (%)
Age (years)			
Median/Range		49/28–76	
Tumor size			
Median/Range		22 mm/5–100 mm	
<20 mm/≥20 mm		41/64	39.0/61.0
Histolopathological features			
Invasive ductal carcinoma		88	83.8
Medullary carcinoma		5	4.8
Invasive lobular carcinoma		2	1.9
Metaplastic carcinoma		2	1.9
Specialized types^a^		8	7.6
T stage			
1/2/3/4/		43/57/2/3/	41/54.3/1.9/2.9
N stage			
01/2/3		65/28/7/5	61.9/26.7/6.7/4.7
Nuclear grade^b^			
1/2/3		2/28/70	1.9/26.7/66.7
Histologic grade^c^			
I/II/III		7/25/68	6.7/23.8/64.8
Ki67 labeling index			
<5%		17	16.2
5% ∼49%		67	63.8
≥50%		21	20
*TP53* gene mutations			
Missense DBM		11	10.5
Missense non-DBM		5	4.8
Frameshift		8	7.6
Nonsense		7	6.7
Splicing		2	1.9

a Adenoid cystic carcinoma, ductal carcinoma in situ, invasive cribriform carcinoma, invasive micropapillary carcinoma, invasive tubular carcinoma, neuroendocrine carcinoma and mucinous carcinoma. ^b,c^ Five samples did not have a defined histologic grade and nuclear grade.

### Mutation analysis of *EGFR*, *KRAS*, *BRAF* and *TP53* genes

The mutation frequency of *KRAS* and *EGFR* gene were 1.9% (2/105) and 1.0% (1/105), respectively. One activating mutation (exon 19 del) was detected using PNA-mediated clamping PCR in a commonly deleted region (codons 746–753) of the *EGFR* gene. Two *KRAS* mutations (G12V and G12D) were identified using PNA-mediated clamping PCR and sequencing. There was no *BRAF*V600E mutation found (Table 3 & Table S1).

**Table 3:** Molecular Characteristics of Triple Negative Breast Cancers (n = 105).

Number of cases (%)	*EGFR* copy number change	*EGFR* mutation	*KRAS* mutation	*TP53* mutation
				
61 (58.1%)	N	−	−	−
25 (23.8%)	N	−	−	**+**
2 (1.9%)	N	−	**+** ^a^	**+**
1 (1.0%)	N	**+** ^b^	−	−
6 (5.7%)	I	−	−	−
5 (4.8%)	I	−	−	**+**
2 (1.9%)	I†	−	−	−
2 (1.9%)	D	−	−	−
1 (1.0%)	D	−	−	**+**
105		1 (1.0%)	2 (1.9%)	33 (31.4%)

'−', wild type; '+', mutant type; 'D', decreased copy number (hemizygous deletion); ‘N’, normal copy number; 'I', increased *EGFR* copy number (single copy gain, amplification and high-copy number amplification); † single exon amplification; ^a^ p.Gly12Val and p.Gly12Asp; ^b^ Deletion on Exon 19. *BRAF* gene mutation was not detected in all cases

### Frequencies and spectrum of *EGFR* gene copy number changes

**Figure 1:**
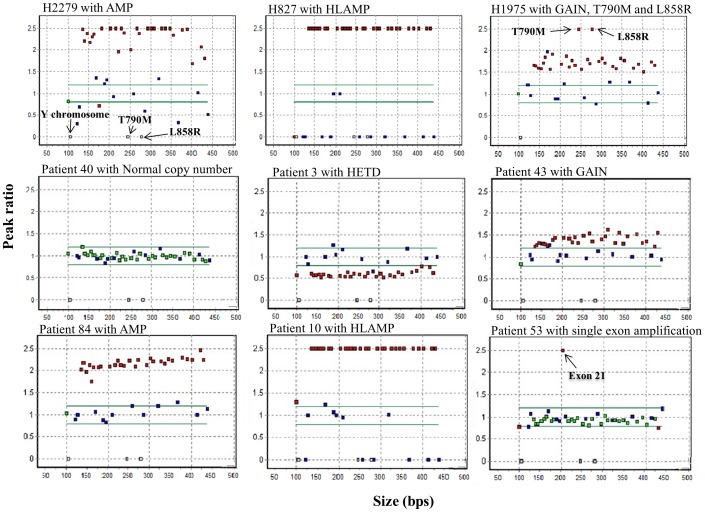
Examples of representative scatter plot patterns of multiplex ligation-dependent probe amplification (MLPA) analysis for the *EGFR* gene. Gain or loss (peak ratio cut off, <0.8 or >1.2) of exons in *EGFR* gene were represented by red squares. Green square indicated within neutral peak ratio of *EGFR* gene. Three white squares indicate specific probe for Y chromosome (105 bps), the L858R (281 bps) and the T790M mutations (246 bps). Blue squares represent control probes. ‘HETD’, hemizygous deletion; ‘GAIN’, copy gain; ‘AMP’, amplifications; ‘HLAMP’, high-copy amplifications.

The frequency of *EGFR* copy number changes was noted in 15.2% of total 105 TNBCs. Among them, increased or decreased EGFR copy number was detected in 13 (12.4%) and 3 cases (2.9%), respectively. Of 13 cases, two cases (1.9%) showed an increased copy number of a single exon (one in exon 1 and the other in exon 21) and 11 cases (10.5%) showed increased copy number changes consists of five single copy gain (GAIN) (4.8%), two amplification (AMP) (1.9%) and four high-copy number amplification (HLAMP) cases (3.8%) (Figure 1 & Table 3).

### Association between TP53 mutations and prognosis in 105 TNBC patients

**Figure 2:**
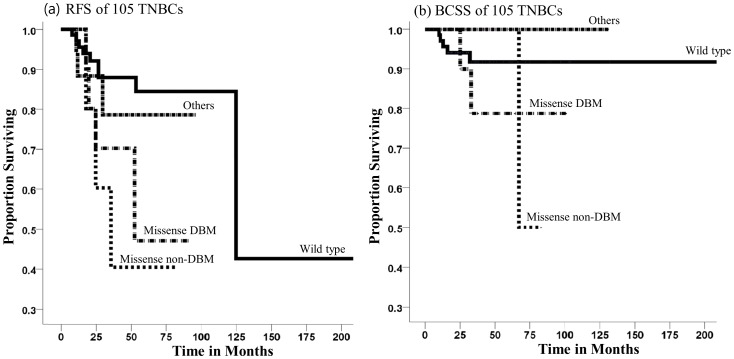
Kaplan-Meier relapse free survival (RFS) and breast cancer specific survival (BCSS) curves of patients with triple negative breast cancer. (a) RFS and (b) BCSS curves stratified by non-mutations, missense mutations in the DBM, missense mutations outside the DBMs and others including nonsense, frameshift and splicing mutations in the *TP53* gene.

Of 105 TNBC patients with a median follow up period of 35 months, there were 20 (19.0%) cases of breast cancer relapse, and 8 cases (7.6%) were due to breast cancer-related death. Kaplan-Meier survival analysis of TNBC patients grouped according to the type of TP53 mutation showed that missense mutations in DBM (*P* = 0.036) and non-DBM (*P* = 0.011) were associated with a higher relapse rate compared with patients without the mutations, whereas other types of mutations including frameshift, nonsense, and splicing mutations (*P* = 0.462) was not significant. The relapse incidence per 100 persons at 25 months for non-mutations, DBM mutations and non-DBM missense mutation groups were 8, 30, 40, respectively (Figure 2A). The DBM mutations (*P* = 0.311), non-DBM missense mutation (*P* = 0.449) and other mutations (*P* = 0.274) were not significantly associated with BCSS compared with wild type (figure 2B). Prognostic factors associated with relapse free survival in the univariate analysis had higher T and N stage and TP53 mutation status. However, in the multivariate analysis, only N stage with grade 2 or 3 was associated with relapse rate (Table 4).

**Table 4:** Univariate and Multivariate analysis of Relapse Free Survival in 105 TNBCs.

	Univariate			Multivariate		
	HR	95% CI of HR	*P*	HR	95% CI of HR	*P*
T status						
T≤1	1			1		
T≥3	6.118	1.381–27.109	0.017	3.648	0.789–16.868	0.098
N status						
N≤1	1			1		
N≥2	4.16	1.481–11.687	0.007	2.945	1.010–8.588	0.048
TP53 mutations						
Wild type	1			1		
Mutations	2.742	1.080–6.960	0.034	2.193	0.852–5.643	0.104

HR, hazard ratio.

### Sensitivity analysis of *EGFR* MLPA

Detection of the *EGFR* amplifications in HCC827 cell line was possible in spiked samples containing 6.25% of HCC827 DNA (Table 5). All five replicates of spiked samples with 6.25% of HCC827 DNA showed *EGFR* amplifications.

**Table 5:** Analytical Sensitivity of MLPA for *EGFR* copy number changes in the HCC827 cell line.

Concentrations of HCC827 (%)^a^	Number of replicates	MLPA
100	3	**+** ^b^
50	3	**+**
25	5	**+**
12.5	5	**+**
6.25	5	**+**
3.13	3	–
Wild type DNA	3	–

MLPA, multiplex ligation-dependent probe amplification. ^a^DNA of the HCC827 was serially diluted with wild type DNA. ^b^ All spiked samples analyzed using MLPA three or five times, and all replicates with from 100% to 6.25% of HCC827 DNA showed *EGFR* amplifications.

## Discussion

Recently, oncogenic mutations linked to the *AKT* and *MEK/MAPK* pathways have been evaluated as possible predictive markers associated with a good response to EGFR-targeted agents in TNBCs [15], [17], [18], [36]. Grob *et. al*., have reported that no mutation was found in *EGFR, KRAS,* and *BRAF* among 63 TNBCs, and only one *EGFR* amplification case was detected [15], consistent with other results in European patients [16], [17]. However, mutations and copy number changes of the *EGFR* gene were more frequently observed in Asian patients with a detection rate up to 11.4% [18] and 21% [19], respectively. Out of all 105 TNBCs, 10.5% showed increased *EGFR* copy number changes including five GAIN, four HLAMP, two AMP, and one case harbored activating mutation (exon 19 del). These genetic aberrations were thought to be susceptible to either EGFR*-*TKIs or EGFR-blocking monoclonal antibody therapy in primary non–small cell lung cancer [9]–[12] or in recurrent glioblastoma [13]. The frequency of activating *EGFR* mutations in our study was lower than that of a recent Asian study among patients in Singapore (8/70). However, the frequencies of activating *EGFR* mutations and copy number changes in our study were comparable to that of a study including Caucasian patients [20], which reported four HLAMP cases (6.2% 4/65) and one *EGFR* gene mutation case (1.5%, 1/65).

In a total of 105 TNBCs, two *KRAS* point mutations and three HETD cases were detected (4.8%, 5/105). Mutations of the *KRAS* gene are known to be negative predictive genetic markers for EGFR targeted therapy and were significantly associated with resistance to EGFR-blocking monoclonal antibody (cetuximab) in colon cancer [27], [37]. In our study, three HETD cases were detected, but a partial deletion variant of *EGFR* gene, EGFRvIII (in-frame deletion of exon 2–7) reported frequently in glioblastoma [13], [38] and breast cancer [39], was not observed. Two cases showed only single exon copy number amplifications. Tang *et al*. showed that EGFRvIII enhanced the tumorigenic potential of breast cancer cells in mice [40] and *Sok et al*. also showed that xenografts expressing EGFRvIII grew more rapidly than tumors derived from vector-transfected control cells [38]. However, the functions of the whole gene deletion of *EGFR* or the single exon copy number amplifications in tumor cells were not evaluated. Therefore, further investigation is needed to characterize the functional and clinical significance of the whole gene deletion of *EGFR* and the single exon copy number changes.

*TP53* mutations were more prevalently detected in tumors with high grade, large size, and node-positivity. The relative risk of deaths with *TP53* mutations was 2 to 3 times higher than those without *TP53* mutations, especially for the group with missense mutation in DBM was associated with shortest survival [23]. In our study, patients with missense mutations showed reduction in relapse free survival, but the effect of the missense mutation in the DBM was not stronger than the missense mutation outside the DBM on breast cancer relapse.

Among 105 TNBCs, 5 of 12 tumors with increased *EGFR* copy number or *EGFR* mutation also had *TP53* mutations (Table 3 & Table S1). Several studies have reported that amplification of *EGFR* and inactivation of *TP53* are associated with sensitivity to anti-EGFR monoclonal antibodies in metastatic colorectal cancer [41], [42]. However, in TNBCs, the *TP53* mutation has been limited as one prognostic marker that can identify tumors with more aggressive behavior [43], [44]. Therefore, it is necessary to further evaluate the correlation between *TP53* mutation and sensitivity of anti-EGFR therapy in TNBCs with *EGFR* amplification or *EGFR* mutation.

The frequency of TNBCs varies by race. In African Americans, up to 55% of breast cancers were TNBCs, which was 2–3 times more frequent than in other racial groups [2], [5]. Among Asian populations, TNBCs account for 8.0 to 24.1% of all breast cancers (24.1% in Korean patients; 18.6% in Chinese patients; 8% in Japanese patients) [3], [4], [6]. Copy number changes of the *EGFR* gene were detected in 6.2% (in Western patients) to 21% (in Asian patients) of TNBCs [19], [20].

Several randomized clinical studies of anti-EGFR therapy reported that fewer than 20% of metastatic TNBCs showed response to anti-EGFR therapy [45], [46] and the use of these therapies for targeted breast cancer treatment has been controversial. However, these studies had not clearly validated the genetic predictive markers that can modulate sensitivity or resistance to anti-EGFR therapy as described in other cancers [9]–[13], [47].

According to previous studies, TKIs may be more promising in specific subsets of patients with activating *EGFR* mutations, whereas EGFR-blocking monoclonal antibodies may be more effective in TNBC cases where the whole *EGFR* gene copy number is increased [47], [48]. Therefore, analysis of *EGFR* mutations and copy number changes could offer new therapeutic options for TNBC patients. Moreover, analysis of *EGFR* mutations and copy number changes may be more beneficial in the racial groups with high frequencies of TNBCs and with *EGFR* gene aberrations.

This study provides the spectrum of copy number changes and mutation statuses of the *EGFR* gene in Korean TNBC patients. Future studies should include evaluation of clinical outcomes of TNBC patients who undergo anti-EGFR therapy according to the genetic status of *EGFR*.

## Supporting Information

Table S1 Forty-four TNBCs with Abnormal Status of *EGFR, KRAS* or/and *TP53*.
(DOCX)
