# Correction: Spectrum of *EGFR* Gene Copy Number Changes and *KRAS* Gene Mutation Status in Korean Triple Negative Breast Cancer Patients

**DOI:** 10.1371/journal.pone.0114660

**Published:** 2014-11-25

**Authors:** The PLOS ONE Staff

There is an error in the funding statement. Please refer to the correct funding statement below.

This study was supported by a faculty research grant from Yonsei University College of Medicine for 2012 (6-2012-0028). The funders had no role in study design, data collection and analysis, decision to publish, or preparation of the manuscript.

## References

[1] Kim Y, Kim J, Lee H-D, Jeong J, Lee W, et al (2013) Spectrum of *EGFR* Gene Copy Number Changes and *KRAS* Gene Mutation Status in Korean Triple Negative Breast Cancer Patients. PLoS ONE 8(10): e79014 doi:10.1371/journal.pone.0079014 24205362 10.1371/journal.pone.0079014 PMC3813621

