# Supplementary material for: Spectrum of EGFR Gene Copy Number Changes and KRAS Gene Mutation Status in Korean Triple Negative Breast Cancer Patients
Source: PLoS One. 2013 Oct 30;8(10):e79014. doi: 10.1371/journal.pone.0079014 (PMC3813621; doi:10.1371/journal.pone.0079014)
Supplement: Table S1 — Forty-four TNBCs with Abnormal Status of EGFR, KRAS or/and TP53. (DOCX) [file pone.0079014.s001.docx]

Table S1. Forty-four TNBCs with Abnormal Status of *EGFR, KRAS* or/and *TP53*

| Case | Age(year) | *EGFR* Mutation | *EGFR* CNVs | *KRAS* Mutation | *TP53* Mutation |
| --- | --- | --- | --- | --- | --- |
| Patient 2 | 65 | WT | WT | WT | p.Arg175his |
| Patient 3 | 32 | WT | HETD | WT | p.Ile255del |
| Patient 4 | 49 | WT | WT | WT | p.Arg175his |
| Patient 6 | 47 | WT | WT | WT | c.560-2A>G |
| Patient 7 | 41 | WT | WT | WT | p.Arg175his |
| Patient 8 | 40 | WT | WT | WT | p.Cys238Tyr |
| Patient 10 | 57 | WT | HLAMP | WT | WT |
| Patient 11 | 28 | WT | WT | WT | p.Leu265Pro |
| Patient 13 | 34 | WT | WT | WT | p.Gly154Val |
| Patient 15 | 42 | WT | WT | WT | p.Leu194His |
| Patient 19 | 45 | WT | WT | WT | p.Arg213* |
| Patient 21 | 41 | WT | GAIN | WT | p.Met237Ile |
| Patient 22 | 39 | WT | WT | WT | p.Cys238Arg |
| Patient 24 | 37 | WT | HETD | WT | WT |
| Patient 25 | 54 | WT | WT | WT | p.Arg196* |
| Patient 26 | 44 | WT | WT | WT | p.Arg213* |
| Patient 27 | 46 | WT | WT | WT | p.Cys242*fs* |
| Patient 29 | 38 | WT | WT | WT | p.Gln331* |
| Patient 31 | 42 | WT | WT | WT | p.Cys242*fs* |
| Patient 32 | 49 | WT | WT | WT | p.Arg273Cys |
| Patient 34 | 39 | WT | WT | WT | p.Tyr220Cys |
| Patient 36 | 43 | WT | WT | WT | c.559+1G>A |
| Patient 38 | 54 | WT | Exon 1^e^ | WT | WT |
| Patient 39 | 40 | WT | WT | WT | p.Arg196* |
| Patient 40 | 73 | WT | WT | p.Gly12Val | p.Phe292*fs* |
| Patient 43 | 28 | WT | GAIN | WT | WT |
| Patient 45 | 64 | WT | GAIN | WT | p.Asn263*fs* |
| Patient 46 | 49 | WT | GAIN | WT | WT |
| Patient 47 | 40 | WT | GAIN | WT | p.Gln136* |
| Patient 49 | 54 | WT | WT | WT | p.Cys141Tyr |
| Patient 52 | 56 | Exon 19 del | WT | WT | WT |
| Patient 53 | 70 | WT | Exon 21 | WT | WT |
| Patient 56 | 40 | WT | WT | WT | p.Arg273Cys |
| Patient 58 | 70 | WT | HLAMP | WT | p.Phe134dup |
| Patient 76 | 46 | WT | HLAMP | WT | WT |
| Patient 82 | 46 | WT | WT | WT | p.Ser241Tyr |
| Patient 84 | 57 | WT | AMP | WT | WT |
| Patient 89 | 46 | WT | HLAMP | WT | WT |
| Patient 90 | 69 | WT | WT | p.Gly12Asp | p.Tyr234His |
| Patient 97 | 50 | WT | WT | WT | p.Gln317*fs* |
| Patient 99 | 61 | WT | HETD | WT | WT |
| Patient 102 | 44 | WT | WT | WT | p.Ala159del |
| Patient 103 | 58 | WT | WT | WT | p.Arg213* |
| Patient 105 | 46 | WT | AMP | WT | p. Ala189Val |

‘CNV’, copy number variation, ‘WT’, wild type, ‘HETD’, hemizygous deletion; ‘GAIN’ copy gain, ‘AMP’, amplifications; ‘HLAMP’, high-copy amplifications
